# Engineering a novel glucose-tolerant β-glucosidase as supplementation to enhance the hydrolysis of sugarcane bagasse at high glucose concentration

**DOI:** 10.1186/s13068-015-0383-z

**Published:** 2015-12-01

**Authors:** Li-chuang Cao, Zhi-jun Wang, Guang-hui Ren, Wei Kong, Liang Li, Wei Xie, Yu-huan Liu

**Affiliations:** School of Life Sciences, Sun Yat-sen University, Guangzhou, 510275 People’s Republic of China; South China Sea Bio-Resource Exploitation and Utilization Collaborative Innovation Center, Sun Yat-sen University, Guangzhou, 510275 People’s Republic of China; State Key Laboratory for Biocontrol, School of Life Sciences, Sun Yat-sen University, Guangzhou, 510275 People’s Republic of China

**Keywords:** β-Glucosidase, Glucose-tolerance, Metagenomic library, Thermostability, Directed evolution, Cellulase, Cellulose refining

## Abstract

**Background:**

Most β-glucosidases reported are sensitive to the end product (glucose), making it the rate limiting component of cellulase for efficient degradation of cellulose through enzymatic route. Thus, there are ongoing interests in searching for glucose-tolerant β-glucosidases, which are still active at high glucose concentration. Although many β-glucosidases with different glucose-tolerance levels have been isolated and characterized in the past decades, the effects of glucose-tolerance on the hydrolysis of cellulose are not thoroughly studied.

**Results:**

In the present study, a novel β-glucosidase (Bgl6) with the half maximal inhibitory concentration (*IC*_50_) of 3.5 M glucose was isolated from a metagenomic library and characterized. However, its poor thermostability at 50 °C hindered the employment in cellulose hydrolysis. To improve its thermostability, random mutagenesis was performed. A thermostable mutant, M3, with three amino acid substitutions was obtained. The half-life of M3 at 50 °C is 48 h, while that of Bgl6 is 1 h. The *K*_cat_/*K*_m_ value of M3 is 3-fold higher than that of Bgl6. The mutations maintained its high glucose-tolerance with *IC*_50_ of 3.0 M for M3. In a 10-h hydrolysis of cellobiose, M3 completely converted cellobiose to glucose, while Bgl6 reached a conversion of 80 %. Then their synergistic effects with the commercial cellulase (Celluclast 1.5 L) on hydrolyzing pretreated sugarcane bagasse (SCB) were investigated. The supplementation of Bgl6 or mutant M3 to Celluclast 1.5 L significantly improved the SCB conversion from 64 % (Celluclast 1.5 L alone) to 79 % (Bgl6) and 94 % (M3), respectively. To further evaluate the application potential of M3 in high-solids cellulose hydrolysis, such reactions were performed at initial glucose concentration of 20–500 mM. Results showed that the supplementation of mutant M3 enhanced the glucose production from SCB under all the conditions tested, improving the SCB conversion by 14–35 %.

**Conclusions:**

These results not only clearly revealed the significant role of glucose-tolerance in cellulose hydrolysis, but also showed that mutant M3 may be a potent candidate for high-solids cellulose refining.

**Electronic supplementary material:**

The online version of this article (doi:10.1186/s13068-015-0383-z) contains supplementary material, which is available to authorized users.

## Background

Biological refining of cellulose is important for the development of alternative energy [1–5]. The efficient biological conversion of cellulose usually requires the synergy of three kinds of enzymes: endoglucanases (EGs, EC 3.2.1.4), exoglucanase (also named cellobiohydrolases, CBHs, EC 3.2.1.91), and β-glucosidases (BGLs, EC 3.2.1.21). Endoglucanases randomly hydrolyze the β-1, 4-glucosidic bond in the non-crystalline area of cellulose, mainly producing dextrin and oligosaccharides. Exoglucanases liberate cellobiose units from cellulose chain end, while β-glucosidases convert cellobiose into glucose [6, 7]. However, most β-glucosidases reported are sensitive to glucose, hence they are easily inhibited by the end product feedback (glucose), leading to the accumulation of cellobiose and oligosaccharide. The accumulated cellobiose and oligosaccharide further inhibit the activities of endoglucanase and exoglucanase, which ultimately blocks the whole process of cellulose degradation. As a result, β-glucosidase has been considered to be the rate limiting enzyme and the bottleneck of efficient degradation of cellulose through enzymatic route [6–8]. Thus, there are ongoing interests in searching for glucose-tolerant β-glucosidases, which are still active at high concentration of glucose.

Although many β-glucosidases with different glucose-tolerances have been isolated from bacteria [9–11], fungi [12, 13], yeast [14], and metagenomic libraries [15, 16], the effects of glucose-tolerance on the hydrolysis of cellobiose or cellulose are not thoroughly studied. For example, a β-glucosidase with the inhibition constant (*K*_i_) of 1.4 M was isolated from *Canada plate*. The hydrolysis rates of 10 % cellobiose (w/v) by this enzyme were similar in the presence or absence of glucose (6 %, w/v) [14], indicating the potential advantage of glucose-tolerant β-glucosidases in cellobiose hydrolysis. The β-glucosidase from *N. crassa* with *K*_i_ of 10.1 mM maintained 31 % activity while the β-glucosidase from C*. globosum* with *K*_i_ of 0.68 mM maintained only about 8 % activity at 400 mM glucose/50 mM cellobiose [17]. A recent research showed that the glucose-tolerant β-glucosidase G1mgNtBG1 from Termite *Nasutitermes takasagoensis* (*K*_i_ value of 0.6 M) was more effective than Novozym 188 (*K*_i_ value less than 0.1 M) at releasing reducing sugars when mixed with Celluclast 1.5 L to degrade Avicel [18]. But these two β-glucosidases showed not only different glucose-tolerance levels, but also different thermostability, kinetic parameters, and substrate specificity [7, 18]. Thus, the better performance of G1mgNtBG1 in the process of Avicel hydrolysis was the result of combined effects of these factors. Accordingly, it is still unclear to what extent the glucose-tolerance of β-glucosidase affects the hydrolysis of cellulose. In order to further understand the role of glucose-tolerance in cellulose hydrolysis, more studies on the β-glucosidases with different glucose-tolerances, their performance in cellulose hydrolysis, and the effect of glucose on their performance during the process are needed.

In this work, a novel glucoside hydrolase family 1 (GH1) β-glucosidase (Bgl6) was isolated from a metagenomic library of Turpan Depression. The recombinant Bgl6 showed excellent glucose-tolerance. The addition of Bgl6 to Celluclast 1.5 L significantly enhanced the glucose production from pretreated sugarcane bagasse (SCB). However, its half-life at 50 °C is only 1 h. Therefore, random mutagenesis was performed to improve its thermostability and a thermostable mutant M3 was obtained. Then the enzymatic properties of the mutants were characterized and compared with that of wild-type (WT). Their hydrolysis rates of cellobiose (10 %, w/v) and synergistic effect with Celluclast 1.5 L on hydrolyzing pretreated SCB (10 %, w/v) were also investigated. To further assess the potential of M3 in the high-solids cellulose hydrolysis, the SCB hydrolysis was performed at different initial glucose concentrations. Results showed that it functioned well at glucose concentration as high as 500 mM.

## Results and discussion

### β-Glucosidase screening and sequence analysis

A plasmid metagenomic library which contained about 260 Mb of metagenomic DNA was successfully constructed for screening novel β-glucosidases. Five positive clones were identified out of nearly 50,000 clones by functional screening, and one of the positive clones was finally selected for further studies due to its high glucose-tolerance (details see below). Sequence analysis of this positive clone revealed an open reading frame (named *bgl6*) of 1371 bp, which encodes a 456-amino-acid protein (Bgl6). A protein blast search (Blastp) showed that Bgl6 has 90 % identity with the β-glucosidase from *Brevundimonas abyssalis* [Genbank accession number: WP_021696816]. A search of Conserved Domains Database (CDD) revealed that Bgl6 is a member of glycoside hydrolase family 1 (GH1). Multiple sequence alignment of Bgl6 with other glucose-tolerant β-glucosidases from GH1 family indicated that they share sequence similarity (Additional file 1: Figure S1). The well-conserved catalytic proton donor and nucleophile in GH1 family, Glu^171^ and Glu^357^ [19], were marked by a “*” in Additional file 1: Figure S1.

### Screening for mutants with improved thermostability

An epPCR library contained about 46,000 colonies was successfully constructed for screening mutants of Bgl6 with improved thermostability. Twenty-five randomly picked clones were sequenced to evaluate the diversity of the library. Results showed that the error rate of this library was 1.9 nucleotide changes/kb. About 37 % of the clones were identified to be active by the black halos formed around the colonies. Then they were transformed to duplicate LB-agar plates containing a low induction concentration of IPTG (0.02 mM) to avoid significant changes of the protein expression. After a 48 h cultivation at 37 °C, one plate was treated for 20 min at 70 °C. The heat treatment completely inactivated the WT and the mutants with enhanced thermostability would show brown halos around the colonies. Four positive clones were identified out of about 17,000 clones (Additional file 1: Figure S2). Sequence analysis confirmed that the mutations were V174A, W174C, A404V, and L441F, respectively. The combination of three such mutations gave rise to the mutant M3 (W174C/A404V/L441F).

### Overexpression, purification, and enzymatic characterization

Bgl6 and the mutants were overexpressed in a soluble protein fraction using *E. coli* BL21 (DE3) (Additional file 1: Figure S3). The recombinant proteins were purified by metal chelation chromatography. Sodium dodecyl sulfate–polyacrylamide gel electrophoresis (SDS-PAGE) analysis revealed that the molecular mass of the recombinant proteins agrees with the predicted size (51.5 kDa) plus the C-terminal fusion tag of 6 × His.

**Fig. 1:**
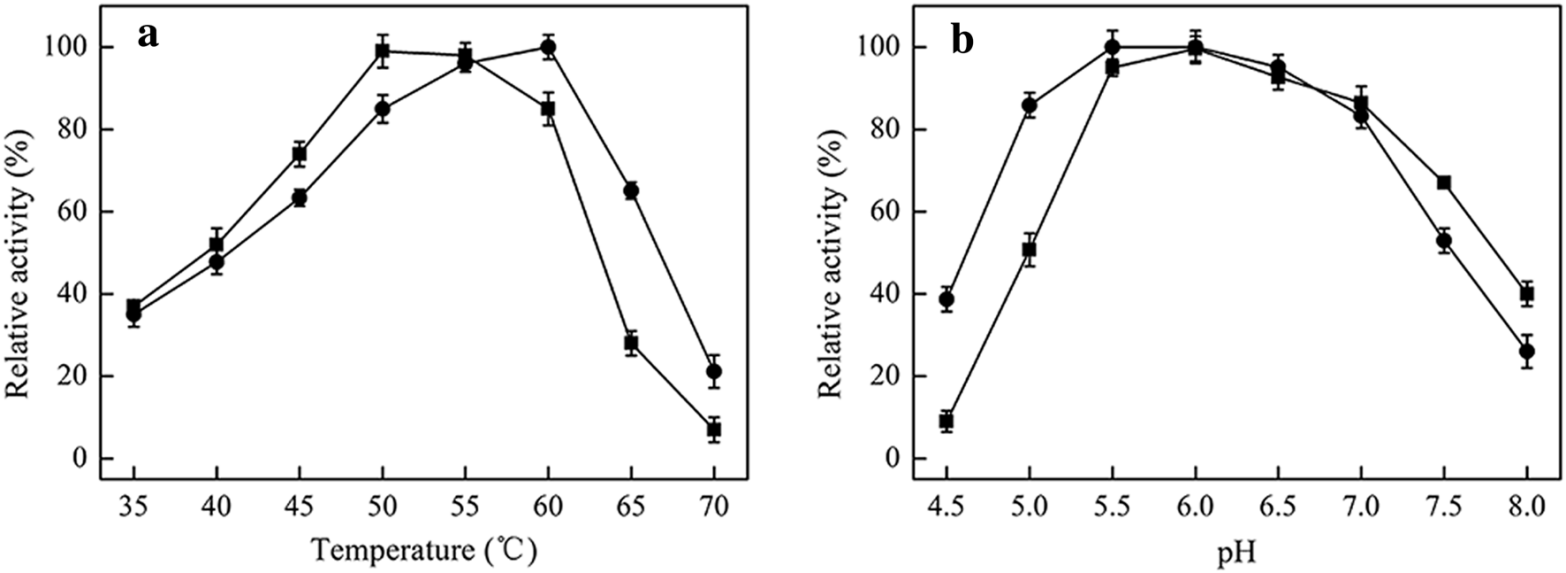
Effects of temperature and pH on the initial reaction rates of Bgl6 (*filled square*) and mutant M3 (*filled circle*). **a** The effects of temperature were measured at 50 °C. **b** The effects of pH were measured at pH 6.0. Data points are the average of triplicate measurements, and *error bars* represent standard deviation

**Fig. 2:**
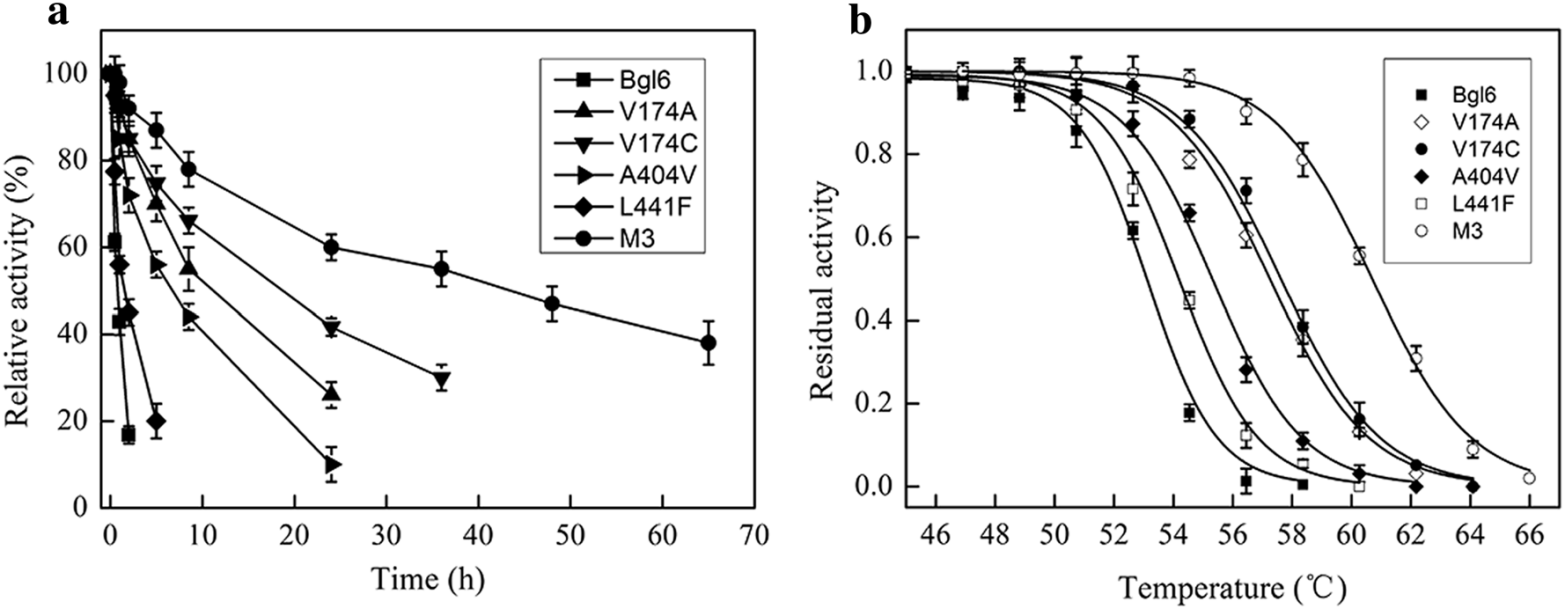
Thermostability of Bgl6 and the mutants. **a** Half-lives of Bgl6 and the mutants at 50 °C are 1 h (Bgl6), 8 h (V174A), 21 h (V174C), 5 h (A404 V), 2 h (L441F), and 48 h (M3). **b** Thermal inactivation curves of Bgl6 and the mutants. The T_50_ values are 53.1 °C (Bgl6), 57.3 °C (V174A), 57.6 °C (V174C), 55.3 °C (A404 V), 54.2 °C (L441F), and 60.7 °C (M3). Data points are the average of triplicate measurements, and *error bars* represent standard deviation

The optimal temperature (T_opt_) of Bgl6 is 50 °C (Fig. 1a). The mutations increase the T_opt_ to 60 °C (M3), which is 10 °C higher than that of the WT (Fig. 1a). At 50 °C, M3 remained about 85 % activity of that at 60 °C. Meanwhile, the optimal pH of the recombinant protein was shifted from 6.0 (Bgl6) to 5.5 (M3) by the mutations (Fig. 1b). This may benefit the utilization of this protein in the practical hydrolysis of cellulose because the optimal pHs of the cellulases employed now are about 5.0 [8, 20, 21]. The half-life of Bgl6 at 50 °C is only 1 h (Fig. 2a). The mutations result in 2–20 folds improvement on this property (Fig. 2a). Combination of three beneficial mutations further extends the half-life to 48 h (M3).

T_50_ determination is a useful method to estimate and directly compare the thermostabilities of enzymes [22, 23]. In line with the results of half-life measurements, the T_50_ values of variants are 1.1–4.5 °C higher than that of WT (Fig. 2b). The T_50_ value of mutant M3 is 60.7 °C, which is 7.5 °C higher than that of the WT (53.1 °C). These results reconfirm the enhanced thermostability of this enzyme.

**Fig. 3:**
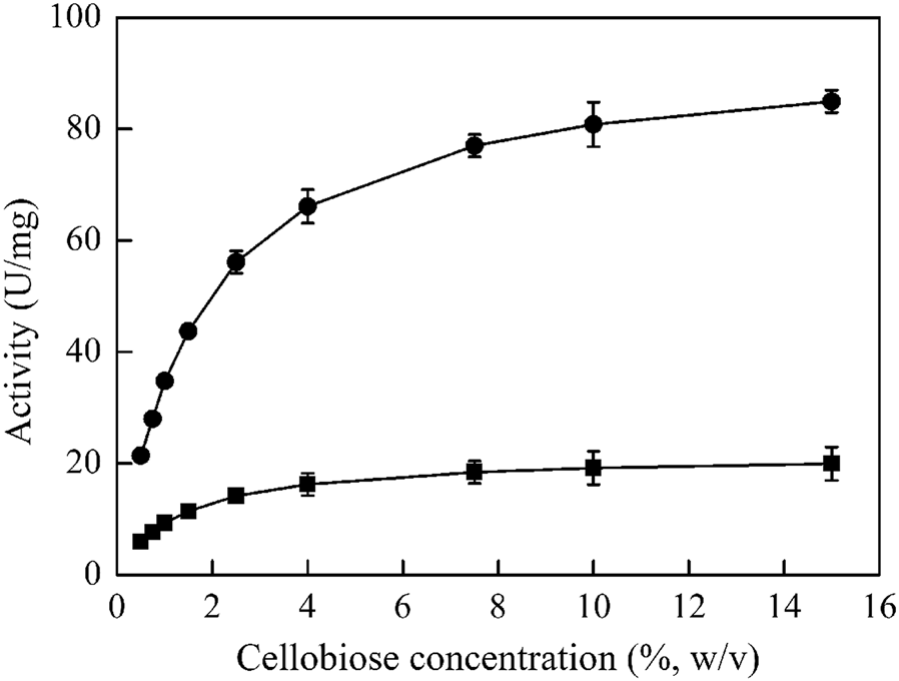
Effects of cellobiose concentration on the initial reaction rates of Bgl6 (*filled square*) and M3 (*filled circle*). The reactions were performed at 50 °C and pH 6.0 with different concentrations of celobiose (0.5–15 %, w/v) as substrate. Data points are the average of triplicate measurements, and *error bars* represent standard deviation

Bgl6 is active towards cellobiose, cellotriose, cellotetrose, and cellopentose (Table 1, Additional file 1: Figure S4). This property is favorable for the usage of this enzyme in cellulose refining as it helps relieve the inhibitory effect of the saccharides to EGs and CBHs during the saccharification process of cellulose [18]. Meanwhile, the recombinant Bgl6 remained highly active towards cellobiose at the concentration of 15 % (w/v, about 440 mM, Fig. 3), and this feature is different from many other β-glucosidases, which are often inhibited by cellobiose at millimolar concentration range. For instance, the β-glucosidase from *Orpinomyces* sp. Strain PC-2 was inhibited when the cellobiose concentration was higher than 1.5 mM [24]; the β-glucosidase from *Trichoderma viride* was inhibited when the cellobiose concentration was higher than 8 mM [25], and the β-glucosidases from *Acremonium thermophilum* (AtBG3), *Thermoascus aurantiacus* (TaBG3), and *Aspergillus sp*. (N188BG) were inhibited when the cellobiose concentration was higher than 5 mM [7]. The substrate inhibition is due to the occurrence of transglycosylation reaction, which is under kinetic control [26, 27]. Although all cellobiose and transglycosylation products will eventually be hydrolyzed to glucose, transglycosylation competes with hydrolysis and thus will hinder the efficient degradation of cellulose [8, 27, 28]. Therefore, the cellobiose-tolerant β-glucosidases, such as Bgl6 and mutant M3 (Fig. 3), may be more suitable under industrial condition where the typical cellobiose concentrations are tens of millimolar [29].

**Table 1:** The substrate specificity of Bgl6

Substrate	Specific activity (U mg^−1^)
pNPG	2.82 ± 0.15
oNPG	37.42 ± 3.34
Cellobiose	21.71 ± 0.27
Cellotriose	17.58 ± 0.17
Cellotetrose	15.84 ± 0.21
Cellopentose	11.95 ± 0.35
Lactose	9.82 ± 0.86
Sucrose	ND
Maltose	ND
Trehalose	2.19 ± 0.04
Salicin	1.78 ± 0.08
CMC	ND
Avicel^ℛ^	ND

*ND* activity not detected after 1 h reaction employing 2 U cellobiose (10 %, w/v) activity

The kinetic parameters of Bgl6 and the mutants were listed in Table 2. The *K*_*m*_ and *K*_cat_/*K*_m_ of Bgl6 are 38.45 mM and 0.56 mM^−1^ S^−1^, respectively. Mutations V174A, V174C, and A404V increased the *K*_cat_ value by 1.5–2 folds while decreasing its affinity to cellobiose. In contrast, mutation L441F increased the affinity towards cellobiose but damaged the catalytic efficiency. As a result, the *K*_cat_/*K*_m_ value of mutant M3 is 3-fold higher than that of Bgl6. According to the report by Teugjas and Väljamäe [7], it seems that high glucose-tolerance is always companied with high *K*_*m*_ value. For example, most of the glucose-tolerant β-glucosidases (with *K*_i_ values about 0.1–1.4 M) have the *K*_*m*_ values between 7 and 70 mM, while their glucose-sensitive homologues (with *K*_i_ values less than 0.1 M) usually have the *K*_*m*_ values between 0.4 and 4 mM [7]. However, in this work, mutation L441F resulted in a lower *K*_*m*_ value (25.91 mM) than that of Bgl6 while keeping a high glucose-tolerance (*IC*_50_ of 3 M). In addition, a recent research showed that the β-glucosidase Bglhi from *Humicola insolens* RP86 was highly glucose-tolerant (*IC*_50_ of about 0.6 M) and affinitive to cellobiose as well (*K*_*m*_ value of 0.38 mM) [12]. These results suggest that it might be feasible to obtain β-glucosidases with the uncommon properties of both high glucose-tolerance and high affinity to cellobiose by protein engineering.

**Table 2:** The kinetic parameters of Bgl6 and the mutants using 10 % cellobiose (w/v) as substrate

Enzymes	Optimal temperature	Optimal pH	*K*_m_ (mM)	*K*_cat_ (s^−1^)	*K*_cat_/*K*_m_(s^−1^ mM^−1^)
Bgl6	50–55	6.0	38.45 ± 1.51	21.50 ± 0.38	0.56
V174A	55–60	6.0	315.39 ± 10.87	35.13 ± 2.46	0.11
V174C	60	5.5	45.07 ± 0.50	48.45 ± 0.39	1.07
A404 V	55	6.5	50.52 ± 2.51	31.97 ± 1.13	0.63
L441F	55	5.5	25.91 ± 2.02	14.73 ± 1.54	0.57
M3	60	5.5	49.19 ± 1.75	83.11 ± 4.12	1.69

The hydrolysis of cellobiose results in two molecules of glucose

The *K*_cat_ and *K*_m_ values were determined on the basis of the Michaelis–Menten equation

### The effects of glucose on the initial reaction rates of Bgl6 and the mutants

**Fig. 4:**
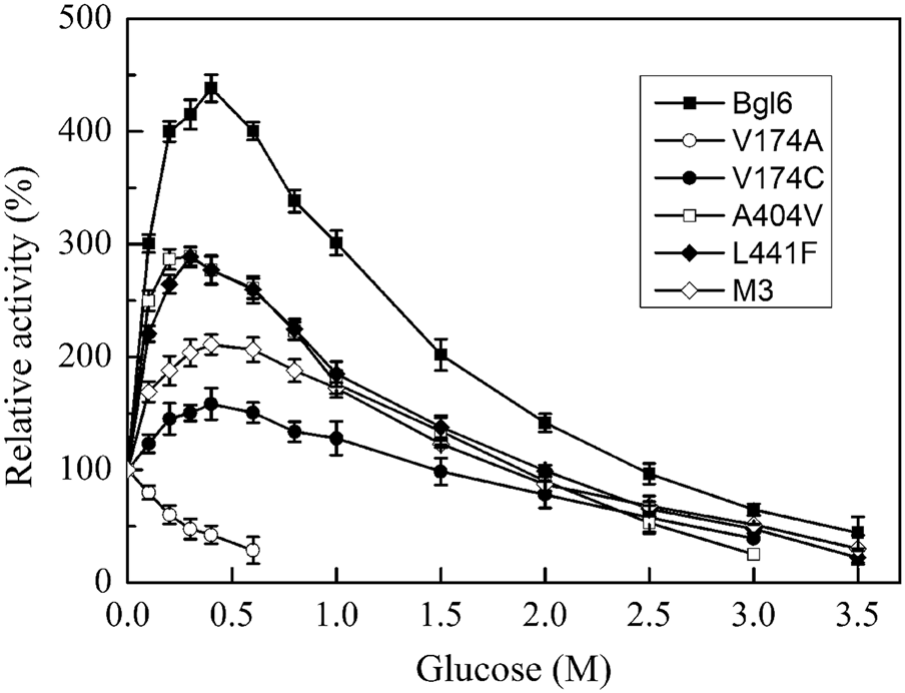
Effects of glucose on the initial reaction rates of Bgl6 and the mutants. The reactions were performed at 50 °C and pH 6.0 with p-Nitrophenyl-β-d-glucopyranoside (pNPG) as substrate. The *IC*_50_ values are 3.5 M (Bgl6), 0.3 M (V174A), 2.5 M (V174C), 2.5 M (A404 V), 3.0 M (L441F), and 3.0 (M3). Data represent the means of three experiments and *error bars* represent standard deviation

Based on the different effects of glucose on the activity, β-glucosidases could be divided into three groups: (1) the β-glucosidases that are strongly inhibited by low concentration of glucose, and most β-glucosidases belong to this group with the *K*_i_ value less than 0.1 M [7, 30]; (2) the β-glucosidases that are tolerant to low concentration of glucose but are inhibited by high concentration of glucose, such as the β-glucosidase from *Aspergillus oryzae* which showed the *K*_i_ of 1.36 M [30], the β-glucosidase from *Candida peltata* which showed the *K*_i_ of 1.4 M [14] and the β-glucosidase from uncultured bacterium which showed the *K*_i_ of 4.28 M [15]; (3) the β-glucosidases that are stimulated by low concentration of glucose and are inhibited by high concentration of glucose. Bgl6 belongs to the last group, whose activity is stimulated more than 4-fold by 0.2–0.6 M glucose (Fig. 4), and this stimulation level was higher than its orthologs from most previous reports. For example, the β-glucosidases from *H. insolens* [12], *S.thermophilum* [13], and *H. grisea* var. *thermoidea* [31] were stimulated more than 2-fold by 0.05–0.2 M glucose; the β-glucosidases from *Bacillus halodurans* C-125 [9], uncultured bacterium [16], and *Neotermes koshunensis* [32] were stimulated about 1.6-, 1.3-, and 1.3-fold by 0.2 M glucose, respectively. The phenomenon of stimulation by glucose seems to be a unique property of GH1 β-glucosidases, which is not observed among the GH3 β-glucosidases until now [33]. The reason for the stimulation could be an allosteric effect by glucose binding to the secondary site [13, 34] or the occurrence of transglycosylation [32]. Therefore, further investigation on the underlying mechanism is required, which leads to better understanding of the catalytic property of β-glucosidase.

The effects of glucose on the activities of the mutants were shown in Fig. 4. The *IC*_50_ values of the mutants were 0.3 M (V174A), 2.5 M (V174C), 2.5 M (A404 V), 3.0 M (L441F), and 3.0 (M3), respectively. The activities of mutants V174C, A404V, L441F, and M3 were stimulated about 1.5-, 2.9-, 2.9-, and 2.1-fold by 0.2–0.5 M glucose, respectively, whereas the activity of mutant V174A was not stimulated by glucose. The active site of GH1β-glucosidases can be divided into three regions: glycone binding site (−1 site), aglycone binding site (+1 site), and substrate entrance site (including +2 site) [35, 36]. Previous research have identified several key amino acids affecting the glucose-tolerance, most of which are located at the substrate entrance site [35, 37]. In this work, residue V174 of Bgl6 is regarded as “aglycone binding site” (Additional file 1: Figure S1), suggesting that the “aglycone binding site” of GH1 β-glucosidases is also involved in glucose-tolerance. In addition, although mutations A404V and L441F did not change the glucose-tolerance (*IC*_50_ value) significantly, they affected the glucose stimulation level of this enzyme (Fig. 4). Residue A404 is next to the well-conserved W405 in GH1β-glucosidases (Additional file 1: Figure S1), and W405 is a part of the glycone binding site (−1 site). Thus, the mutation A404V may change the shape and electrostatic properties of the active site, which play an important role in determining glucose-tolerance [33]. However, the effect of mutation L441F on glucose-tolerance is more difficult to explain because SWISS-MODEL showed that residue L441 is on the surface of the protein and far away from the active site (Additional file 1: Figure S5) [38]. To date, the structural basis for glucose-tolerance is still elusive. Consequently, subsequent study on these key amino acids, such as saturation mutagenesis and structure–activity relationship, may enrich our knowledge about this issue.

### Hydrolysis of cellobiose and SCB by Bgl6 and M3

**Fig. 5:**
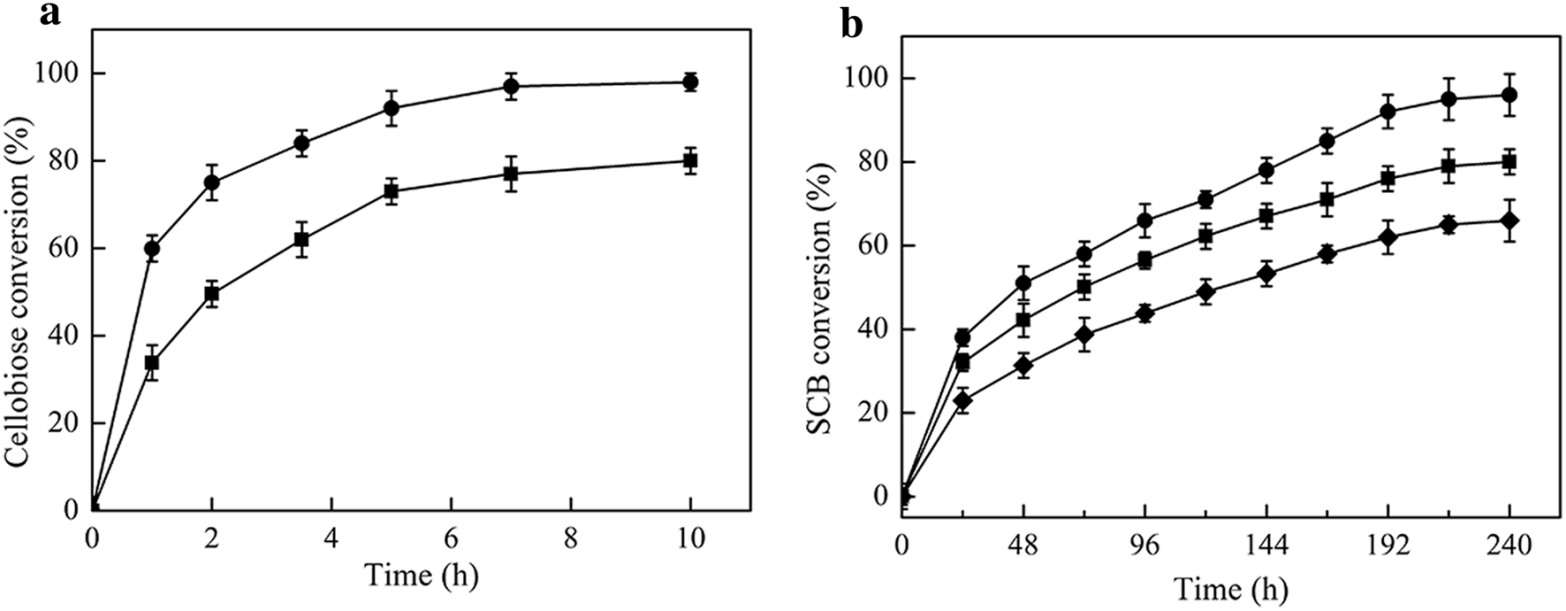
Hydrolysis of cellobiose and pretreated SCB by Bgl6 and mutant M3. The reactions were performed at 50 °C in 100 mM phosphate buffer (pH 6.0). The concentrations of the substrates were 10 % (w/v). **a** Hydrolysis of cellobiose by Bgl6 (*filled square*) and mutant M3 (*filled circle*). **b** Celluclast 1.5 L (*filled diamond*) was used alone as a control. Addition of Bgl6 (*filled square*) and mutant M3 (*filled circle*) to Celluclast 1.5 L improved the SCB conversion. Data represent the means of three experiments and *error bars* represent standard deviation

Compared with Bgl6, mutant M3 has better thermostability, higher *K*_cat_/*K*_m_ value, similar glucose-tolerance but lower affinity to cellobiose. All these enzymatic properties can significantly affect their performance in the hydrolysis of cellobiose and cellulose. To evaluate their efficiency in cellobiose hydrolysis, cellobiose (10 %, w/v) was hydrolyzed at 50 °C in 100 mM phosphate buffer (pH 6.0). The enzyme/substrate ratio was 5 mg/g cellobiose (1:200). As shown in Fig. 5a, mutant M3 completely converted cellobiose to glucose in 10 h of reaction. Whereas, Bgl6 hydrolyzed cellobiose more slowly, reaching about 80 % conversion in the same time. Then the hydrolysis of pretreated sugarcane bagasse (10 % w/v, dry basis) was performed under the same reaction condition. Celluclast 1.5 L was used alone as a control and the β-glucosidase/substrate ratio was 0.5 mg/g SCB (1: 2000). The cellulose content of the pretreated SCB was determined to be 45.7 % (w/w). The time course of the pretreated SCB conversion was shown in Fig. 5b. In a hydrolysis of 240 h, the concentration of the glucose released by Celluclast 1.5 L alone was 3.25 % (w/v), representing 64 % of the total cellulose. The supplementation of Bgl6 or mutant M3 to Celluclast 1.5 L significantly improved the conversion to 79 % (Bgl6) and 94 % (M3), respectively. These results show the advantage of mutant M3 over Bgl6 in the hydrolysis of both cellobiose and cellulose. In addition, the cellobiose concentrations during the SCB hydrolysis were monitored and quantified by HPLC (Additional file 1: Figure S6). When only Celluclast 1.5 L was mixed with SCB, the cellobiose concentration was about 0.51 % (w/v, about 15 mM) at the reaction time of 240 h. The addition of Bgl6 and mutant M3 decreased the concentration to 0.34 % (w/v, about 10 mM, Bgl6) and 0.19 % (w/v, about 6 mM, M3) (Additional file 1: Figure S7). Thin-layer chromatography (TLC) analysis of the hydrolysis products showed that the cello-oligosaccharide concentrations were also lower with supplementation of Bgl6 or M3 than the control (Additional file 1: Figure S8). Lower concentrations of cellobiose and cello-oligosaccharide mean weaker inhibition of EGs and CBHs, which is beneficial to the whole reaction process and thus improves the SCB conversion.

### Effects of glucose on the SCB hydrolysis

**Fig. 6:**
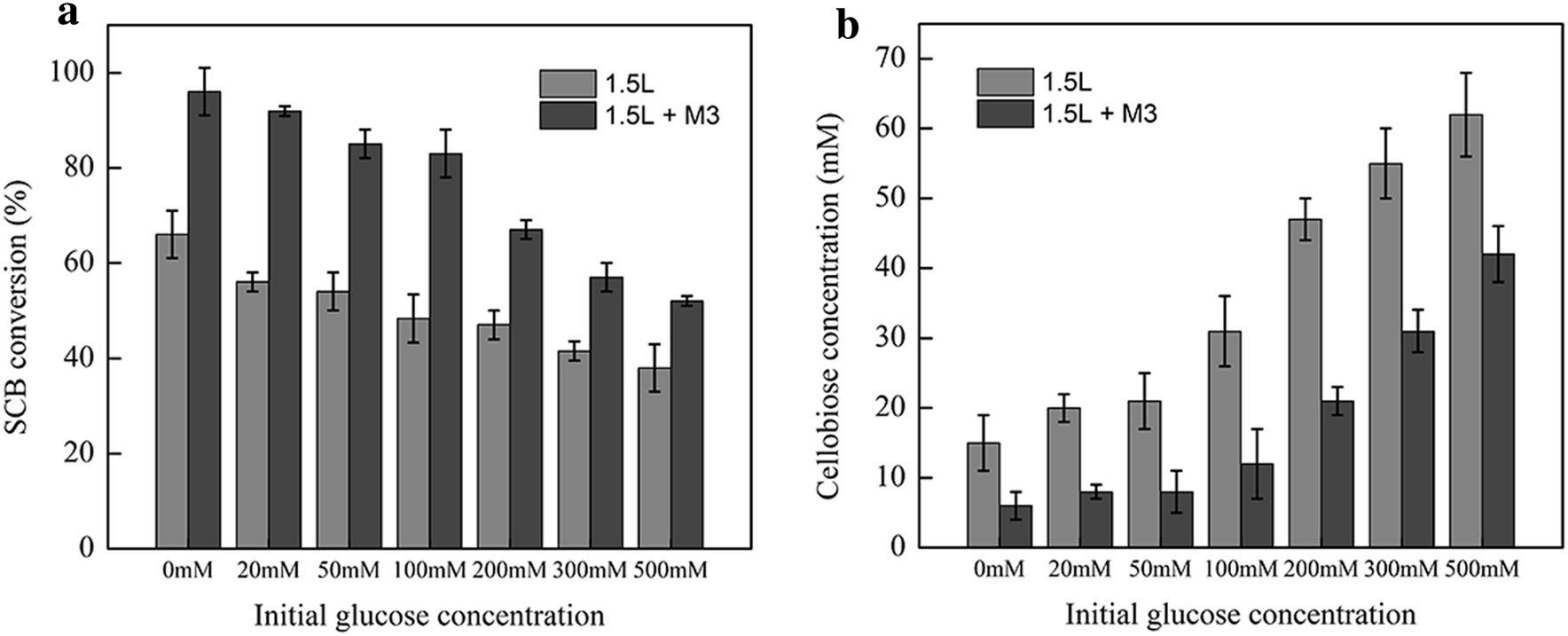
Effects of glucose on the SCB conversions (**a**) and cellobiose concentrations during the reaction (**b**). The reactions were performed at 50 °C in 100 mM phosphate buffer (pH 6.0). The concentration of the substrate was 10 % (w/v). Addition of mutant M3 to Celluclast 1.5 L significantly improved the SCB conversions and decreased the cellobiose concentrations in the hydrolysis. Data represent the means of three experiments and *error bars* represent standard deviation

To further assess the application potential of mutant M3 in the practical hydrolysis where glucose concentration is high, the hydrolysis of 10 % (w/v) pretreated SCB by mutant M3 (with Celluclast 1.5 L) was performed with initial concentration of 20–500 mM glucose. Celluclast 1.5 L was used alone with corresponding initial concentrations of glucose as the control group. The addition of 20 mM glucose in the reaction mixture dramatically decreased the SCB conversion by 9 % (from 64 to 55 %), suggesting that the β-glucosidases in Celluclast 1.5 L are very sensitive to glucose. With the increase of the initial glucose concentration, the conversions gradually declined to about 38 % at 500 mM (Fig. 6a). The supplementation of mutant M3 enhanced the glucose production from SCB under all the conditions tested (Additional file 1: Figure S9), improving the SCB conversion by 14–35 % (Fig. 6a). Meanwhile, the analysis of the cellobiose concentrations during the reaction processes showed that it had a negative correlation with the SCB conversions and a significant decline after the addition of M3 to the reactions (Fig. 6b). These results showed that mutant M3 functioned well at high concentration of glucose, reduced the cellobiose concentrations in the reaction mixture and relieved the inhibition of EGs and CBHs.

To reduce cost, the cellulose hydrolysis must be conducted at high solid loads, which results in high concentrations of hydrolysis products. In the practical hydrolysis process, glucose concentration could reach several hundreds of millimolar [29]. Under this condition, although high catalytic efficiency (*K*_cat_/*K*_m_) is still an attractive property of the β-glucosidases that intend for supporting cellulases in cellulose hydrolysis, the priority is given to the β-glucosidases with higher glucose-tolerance [7]. To date, most of the β-glucosidases employed in the hydrolysis of cellulose belong to GH3 family because of their high *K*_cat_/*K*_m_ values (can be over 100 mM^−1^ S^−1^) [7, 17, 39–41]. However, they are often inhibited by glucose and cellobiose easily [7, 17]. In contrast, some GH1 β-glucosidases are tens or even hundreds folds more glucose-tolerant than the former, but they usually have moderate *K*_cat_/*K*_m_ values (1–13 mM^−1^ S^−1^) [11, 16, 42–44]. So an “ideal” β-glucosidase may be obtained through protein engineering either by enhancing the glucose-tolerance of GH3 β-glucosidases or by improving the *K*_cat_/*K*_m_ values of GH1 β-glucosidases. It has been shown that the success of protein engineering greatly depends on the efficient screening methods [45, 46]. Considering that the mutants with varied glucose-tolerance are difficult to screen for in a general sense for now [22], it would be much easier to screen for mutants with improved activities and then check the glucose-tolerance afterwards [47, 48].

Although mutant M3 can hydrolyze cellobiose efficiently and improve the SCB conversion at glucose concentration as high as 500 mM, the low affinity to cellobiose (*K*_m_ of 49.19 mM) may hinder its employment in the practical application. The main component of CBHs in cellulase is often inhibited by cellobiose with the *IC*_50_ values of a few millimolars [49]. However, a typical cellobiose concentration in high-solids enzymatic cellulose hydrolysis is about 50 mM [29]. To make the CBHs fully functioning, the cellobiose concentration must be maintained at a few tenths millimolars, meaning that the *K*_m_ values of the β-glucosidases operate under this condition should be in the same range or even lower. Thus, the subsequent study on this enzyme should be focused on decreasing its *K*_m_ value by one to two orders of magnitude, which would also dramatically enhance its catalytic efficiency (*K*_cat_/*K*_m_).

## Conclusions

A novel β-glucosidase (Bgl6) was isolated from a metagenomic library with *IC*_50_ of 3.5 M glucose. Its thermostability was significantly enhanced by the substitutions of three amino acids. The mutations also improved the catalysis efficiency by 3-fold and maintained its high glucose-tolerance (*IC*_50_ of 3.0 M). During a 10-h hydrolysis of cellobiose (10 %, w/v), M3 completely converted cellobiose to glucose while Bgl6 reached a conversion of 80 %. The addition of Bgl6 or M3 to Celluclast 1.5 L significantly increased the SCB conversion from 64 % (Celluclast 1.5 L alone) to 79 % (Bgl6) and 94 % (M3), respectively. Furthermore, at initial glucose concentration of 20–500 mM, the supplementation of mutant M3 still improved the SCB conversion by 14–35 %. These results showed the application potential of mutant M3 in high-solids cellulose hydrolysis, and may also provide useful information for the protein engineering of this kind of enzyme.

## Methods

### Materials and strains

*E. coli* DH5α and pUC118 (TaKaRa, Dalian, China) were used for construction of metagenomic libraries, random mutagenesis libraries, and gene cloning. *E. coli* BL21 (DE3) and pET-32a (+) (Novagen, Madison, WI, USA) were used for protein expression. Restriction endonucleases, DNA polymerase, and T4 DNA ligase were purchased from Thermo Fisher Scientific (Hudson, NH, USA). Cellobiose, glucose, and p-nitrophenyl-β-d-glucopyranoside (pNPG) were purchased from Sigma-Aldrich (St. Louis, MO, USA). All other chemicals and reagents were of analytical grade and purchased from commercial sources, unless indicated otherwise.

### Construction and screening of metagenomic library

Topsoil samples (5–10 cm depth) were collected from Turpan Basin (42°51′20′′N, 89°3′19′′E), Xinjiang Uygur Autonomous Region of China and stored at −80 °C until the DNA extraction was performed. The total genomic DNA was extracted by using QIAamp DNA stool Mini kit according to the product manual (QIAGEN, Hilden, Germany). The genomic DNA was then partially digested with *Bam*H I and the DNA fragments of 2.0–8.0 kb were purified by using a QIAquick Gel Extraction Kit (QIAGEN) and inserted into the pUC118 vector, which had been previously digested with *Bam*H I and dephosphorylated with calf intestine alkaline phosphatase (CIAP). The recombinant plasmid was transformed into *E. coli* DH5α via electroporation and the transformants were cultured on LB-agar plates containing 0.1 % (w/v) esculin, 0.25 % (w/v) ferric ammonium citrate and 100 μg/ml ampicillin at 37 °C overnight. Positive colonies were identified by the black halos formed around the colonies [50].

### Directed evolution of Bgl6 for increased thermostability

The plasmid pUC118-*bgl6* was used as the template for the mutagenesis. The random mutagenesis was performed using GeneMorph II Random Mutagenesis Kit (Stratagene, La Jolla, CA, USA) according to the manufacture’s protocol with the primers 5′-GAATT CGAGCTCGGTACCCGGGGATCCATG-3′ (forward) and 5′-GTTGTAAAACGACGGCCAGTGCCAAGCTT-3′ (reverse). The error-prone PCR (epPCR) product was recovered and digested with *Bam*H I and *Hin*d III, and then ligated into pUC118, which had been previously digested with the same restriction enzymes. The ligation product was transformed into *E. coli* DH5α via electroporation. The transformants were cultured on LB-agar plates containing 0.1 % (w/v) esculin, 0.25 % (w/v) ferric ammonium citrate, and 100 μg/ml ampicillin at 37 °C overnight. Single colonies that formed a black halo were picked, transferred on duplicate LB-agar plates containing 100 μg/ml ampicillin and 0.02 mM IPTG. After being cultivated at 37 °C for 48 h, one plate was incubated for 20 min at 70 °C. After the plate was cooled to room temperature, about 5 ml mixture containing 0.5 % (w/v) esculin and 1.0 % (w/v) ferric ammonium citrate was added to it. Positive colonies expressing the mutants with improved thermostability were confirmed by the brown halos formed around the colonies. The corresponding plasmid was subsequently extracted and analyzed by sequencing.

### Site-directed mutagenesis

In vitro site-directed mutagenesis of the *bgl6* gene on plasmid pET-32a (+) was performed by using TaKaRa MutanBEST Kit (TaKaRa, Dalian, China) following the instructions of the manufacturer. The primers used were listed in Additional file 1: Table S1. The correctness of the mutants was confirmed by sequencing.

### Overexpression and purification of the target proteins

The target proteins were overexpressed in *E. coli* BL21 (DE3) using pET32a (+) as vector. The induction was triggered by adding isopropyl-β-d-1-thiogalactopyranoside (IPTG) into the culture at the final concentration of 0.8 mM when the OD_600_ was 0.85. After an additional incubation at 25 °C, 200 rpm for 12 h, cells were collected by centrifugation. The purification was performed by using a His Bind Purification Kit (Novagen) according to the product manual. The purified enzyme in the elution buffer (1 M imidazole, 0.5 M NaCl, 20 mM Tris–HCl, pH 7.9) was further dialyzed three times in phosphate buffer (100 mM, pH 6.0), and then stored at 4 °C for further experiments.

The molecular mass of the denatured recombinant protein was determined by using sodium dodecyl sulfate polyacrylamide gel electrophoresis (SDS-PAGE) with suitable size of protein markers (Thermo Fisher Scientific, Waltham, MA, USA) as standards. The protein concentration was determined by using CoomassiePlus^TM^ (Bradford) Assay Kit (Thermo Fisher Scientific, Waltham, MA, USA) according to the product manual.

### Enzymatic assay

The enzyme activity was determined by using cellobiose as substrate. Appropriately diluted enzyme solution (50 μL) was added into 450 μL of 10 % (w/v) cellobiose solution (100 mM phosphate buffer, pH 6.0). After being incubated for 10 min at 50 °C, the reaction mixture was boiled for 10 min to terminate the reaction. The concentration of glucose released from cellobiose was determined by using a Glucose Oxidase–Peroxidase Assay Kit (Sigma-Aldrich). One unit of enzyme activity was defined as the amount of enzyme required to release 1 μmol of glucose per min.

The activities of Bgl6 towards other substrates were also assayed at 50 °C in 100 mM phosphate buffer (pH 6.0) for 10 min except for Carboxymethyl cellulose (CMC) and Avicel^®^, which were incubated for 1 h. The substrate concentrations were 5 mM (chromogenic substrates), 10 % (w/v, disaccharide and oligosaccharides), and 2 % (w/v, CMC and Avicel^®^), respectively. The total reaction volume was 500 μl for chromogenic substrates, disaccharide, CMC, and Avicel^®^. For oligosaccharides, the total reaction volume was 20 μl. The reaction was stopped by adding 500 μl of 1.0 M sodium carbonate into the reaction solution (chromogenic substrates) or boiling the reaction solution for 10 min (disaccharide, oligosaccharides, CMC and Avicel^®^). The concentration of pNP was determined by measuring the absorbance of the solution at 405 nm. The amount of glucose liberated during the reaction was measured by using a Glucose Oxidase–Peroxidase Assay Kit (Sigma-Aldrich).

### Effects of pH and temperature on the initial reaction rates

The optimal pHs of Bgl6 and the mutants were determined by using cellobiose (10 %, w/v) as substrate in the pH range of 5.0–8.0 at 50 °C. The pH buffers included 100 mM citric acid-sodium citrate buffer (pH 5.0–6.0) and 100 mM phosphate buffer (pH 6.0–8.0). The optimal temperatures of Bgl6 and the mutants were determined by measuring initial reaction rates in the temperature range of 35–65 °C in phosphate buffer (100 mM, pH 6.0).

The thermostability of Bgl6 and the mutants were measured by two parameters. One is half-life (t_1/2_), which was measured by residual activity analysis after incubating the purified enzyme (0.1 mg/ml) for various times intervals at 50 °C. T_1/2_ is defined as the incubation time required inactivating 50 % of the initial enzyme activity. The other one is T_50_, which is defined as the temperature where 50 % of the protein is inactivated in 10 min. Samples containing 0.1 mg/ml purified enzymes (100 mM phosphate buffer, pH 6.0) were inactivated at different temperatures (typically 45–70 °C) for 10 min. After heat treatment, the residual activity was quantified. The T_50_ value was determined by fitting a shifted sigmoid function to the thermal inactivation curves.

### Determination of kinetic parameters

The kinetic parameters (*K*_cat_ and *K*_m_) of Bgl6 and the mutants were determined by assaying the enzymatic activity in 100 mM phosphate buffer (pH 6.0) at 50 °C with seven different concentrations (0.5–5.0 × *K*_m_) of cellobiose. The Michaelis–Menten equation was used to fit all kinetic data to Lineweaver–Burk, and SigmaPlot software (Systat Software, Chicago, IL, USA) was applied to calculate the kinetic parameters.

### Effect of glucose on the initial reaction rates of Bgl6 and the mutants

The effect of glucose on the initial reaction rates was determined according to the method reported by Pei et al. [11]. In brief, the initial reaction rates of β-glucosidase were determined in the presence of increasing concentration of glucose in 100 mM phosphate buffer (pH 6.0) at 50 °C using 5 mM p-nitrophenyl-β-d-glucopyranoside (pNPG) as substrate. The initial reaction rate of enzyme without glucose was considered as 100 %. The half maximal inhibitory concentration (*IC*_50_) was defined as the concentration of the glucose that inhibited 50 % of the initial reaction rates.

### The hydrolysis of cellobiose by Bgl6 and mutant M3

Effects of cellobiose concentration on the initial reaction rates of Bgl6 and mutant M3 were determined under the standard enzymatic assay using 0.5–15 % (w/v) cellobiose as substrate. The amount of glucose released was quantified by using a Glucose Oxidase–Peroxidase Assay Kit (Sigma-Aldrich).

The hydrolysis of 10 % (w/v) cellobiose by Bgl6 and mutant M3 was carried out at 50 °C in 100 mM phosphate buffer (pH 6.0). The reaction was started by adding the purified enzyme into the substrate solution with the enzyme load of 0.5 mg/ml. Samples at different time intervals were collected, boiled for 10 min, filtered and analyzed by high-performance liquid chromatography (HPLC) according to the method of Li et al. [51].

### Effects of glucose on the hydrolysis of pretreated sugarcane bagasse

Sugarcane bagasse (SCB) was kindly provided by Bioengineering Institute of Guangdong General Research Institute for Industrial Technology and pretreated on the basis of the method described by Li et al. [52]. In brief, the SCB was ground and the fractions passed a 40-mesh sieve were collected. The pretreatment was performed in an autoclave for 60 min at 121 °C by using 2 % (w/v) of NaOH with the solid loading of 10 % (w/v). Then the pretreated solids were washed by using hot deionized water until the pH was about 7.0. After being oven-dried at 80 °C, the solid was used for the subsequent experiments.

The reactions were performed at 50 °C in 20 ml of 100 mM phosphate buffer (pH 6.0) shaking at 120 rpm. The substrate concentration and enzyme loads for Celluclast 1.5 L (cellulase from *Trichoderma reesei* ATCC 26921, Sigma-Aldrich) were 10 % (w/v) and 40 filter paper unite (FPU) per gram of SCB by reference to the report of Borges et al. [21]. The enzyme loads for Bgl6 and mutant M3 were 0.5 mg/g SCB. Nystatin and tetracycline were added into the reaction mixture at the final concentration of 80 and 60 μg/ml to avoid contamination. Samples were collected at different time intervals, boiled for 10 min, centrifuged and then the supernatant was used for sugar analysis. The concentration of glucose and cellobiose during the reaction was quantified by HPLC according to method of Li et al. [51].

The reactions to determine the effects of glucose on the SCB hydrolysis were performed according to the same method with the addition of glucose at the final concentration of 20–500 mM before the trigger of the reactions.

The cellulase activity of Celluclast 1.5 L was determined by following the NREL method [53] in 100 mM phosphate buffer (pH 6.0). The SCB conversion was calculated according to the following formula:

$$ {\text{SCB conversion}}\text{ }(\% ) = \frac{{{\text{Glucose concentration}}\text{ }{\rm (g/L)}}}{{{\text{Solid loading}} \times {\text{cellulose }}\% \times 1.11}} \times 100\;\% $$

where glucose concentration referred to the glucose released from SCB, which equaled to the total glucose concentration minus the initial glucose concentration; cellulose (%) was determined based on the NREL method [54] and a new correction method [55]; 1.11 was the coefficient of glucose converted from cellulose; solid loading was 100 g/L in this study.

### Statistical analysis

All experiments in this study were conducted at least three times and all data were treated by analysis of variance (ANOVA) by using IBM SPSS (Statistical Package for the Social Sciences, IBM SPSS. Inc., Chicago, IL, USA). Significant differences were defined as *P* < 0.05.

### Nucleotide sequence accession number

The nucleotide sequence of *bgl6* has been submitted to GenBank, and its accession number is KP736171.

## Additional file

10.1186/s13068-015-0383-z **Figure S1.** Multiple sequence alignment of Bgl6 with other glucose tolerant β-glucosidases from GH1 family. **Figure S2.** Positive clones with improved thermostability. **Figure S3.** SDS-PAGE analysis of the recombinant Bgl6 and the mutants. **Figure S4.** Thin-Layer Chromatography (TLC) analysis of the hydrolysis of cellobiose and cello-oligosaccharide by Bgl6. **Figure S5.** Positions of the mutations in Bgl6. **Figure S6.** High-Performance Liquid Chromatography (HPLC) analysis of the concentration of the celllobiose released from SCB. **Figure S7.** Time course of the cellobiose concentrations in the SCB hydrolysis. **Figure S8.** Thin layer chromatography (TLC) analysis of the hydrolysis of hydrolysis products from SCB. **Figure S9.** Effects of glucose on the hydrolysis of pretreated SCB (10 %, w/v) by Celluclast 1.5 L (♦) alone and supplemented with mutant M3 (●). **Table S1.** Primers used to construct the mutants of Bgl6.

## Abbreviations

*E. coli*: *Escherichia coli*
CIAP: Calf intestine alkaline phosphatase
IPTG: isopropyl-β-d-1-thiogalactopyranoside
SDS-PAGE: sodium dodecyl sulfate–polyacrylamide gel electrophoresis
pNPG: p-Nitrophenyl-β-d-glucopyranoside
oNPG: o-Nitrophenyl-β-d-galactopyranoside
CMC: carboxymethyl cellulose
TLC: thin-Layer Chromatography
HPLC: high-performance liquid chromatography
