# Supplementary material for: Engineering a novel glucose-tolerant β-glucosidase as supplementation to enhance the hydrolysis of sugarcane bagasse at high glucose concentration
Source: Biotechnol Biofuels. 2015 Dec 1;8:202. doi: 10.1186/s13068-015-0383-z (PMC4666061; doi:10.1186/s13068-015-0383-z)
Supplement: Supplementary file 1 — 10.1186/s13068-015-0383-z Figure S1. Multiple sequence alignment of Bgl6 with other glucose tolerant β-glucosidases from GH1 family. Figure S2. Positive clones with improved thermostability. Figure S3. SDS-PAGE analysis of the recombinant Bgl6 and the mutants. Figure S4. Thin-Layer Chromatography (TLC) analysis of the hydrolysis of cellobiose and cello-oligosaccharide by Bgl6. Figure S5. Positions of the mutations in Bgl6. Figure S6. High-Performance Liquid Chromatography (HPLC) analysis of the concentration of the celllobiose released from SCB. Figure S7. Time course of the cellobiose concentrations in the SCB hydrolysis. Figure S8. Thin layer chromatography (TLC) analysis of the hydrolysis of hydrolysis products from SCB. Figure S9. Effects of glucose on the hydrolysis of pretreated SCB (10 %, w/v) by Celluclast 1.5 L (♦) alone and supplemented with mutant M3 (●). Table S1. Primers used to construct the mutants of Bgl6. [file 13068_2015_383_MOESM1_ESM.doc]

**Additional materials**

1. **Additional Figures**

**1.1 Figures Captions**

**Additional Figure S1.** Multiple sequence alignment of Bgl6 with other glucose tolerant β-glucosidases from GH1 family. Sequence alignment was performed by Clustal X2.0. The catalytically important amino acids were indicated as * on the top of the alignment. The amino acids mutated in this study were indicated as ↓. The residues involved in the glycine binding site, aglycine binding site and entrance of the active site were indicated as number 1, 2 and 3 respectively on the bottom of the alignment according to Lee et al. [1]. AAA25311, bgl B from *Thermobispora bispora*; ACY09072, uncultured bacterium; ADD96762, uncultured bacterium; AFN70956, Bgl1269 from uncultured bacterium; BAA74958, *Humicola grisea* var. *thermoidea*; BAB91145, *Neotermes koshunensis*; BAI50022.1, G1mgNtBG1 from *Nasutitermes takasagoensis*; CCA60742, Fibgl1A from *Fervidobacterium islandicum*; NP_242789, Bhbgl from *Bacillus halodurans* C-125; YP_003852393, *Thermoanaerobacterium Thermosaccharolyticum* DSM 571.

**Additional Figure S2.** Positive clones with improved thermostability.

**Additional Figure S3.** SDS-PAGE analysis of the recombinant Bgl6 and the mutants. (A) Lane M, standard protein molecular mass marker (Thermo Fisher Scientific, sizes in kilodaltons are indicated on the left); Lane 1, the whole-cell debris of Bgl6; Lane 2, the supernatant of cell lysate of Bgl6; Lane3, the purified Bgl6 recombinant protein by Ni^2+^ affinity chromatography. (B) Lane M, standard protein molecular mass marker; Lines 1-5, the supernatant of cell lysate of the mutants: Line 1, V174A; Line 2, W174C; Line 3, A404V; Line 4, L441F; Line 5, M3; Lines 6-10, the purified recombinant protein of the mutants: Line 6, V174A; Line 7, W174C; Line 8, A404V; Line 9, L441F; Line 10, M3.

**Additional Figure S4.** Thin-Layer Chromatography (TLC) analysis of the hydrolysis of cellobiose and cello-oligosaccharide by Bgl6. Butanol-ethanol-water (5:3:2, v/v/v) was used as the mobile phase, and visualization was achieved by spraying a solution containing 20 % (v/v) sulfuric acid and 0.5 % (w/v) 3, 5-dihydroxytoluene to a plate (TLC Silica gel 60 F254, Merck, Germany) and heating it at 85 °C for 15 min. Lane M1, glucose; Lane M2, cellobiose; Lane M3, cellotriose ; Lane M4, cellotetrose ; Lane 1, the hydrolysis of cellobiose by Bgl6; Lane 2, the hydrolysis of cellotriose by Bgl6; Lane 3, the hydrolysis of cellotetrose by Bgl6.

**Additional Figure S5.** Positions of the mutations in Bgl6. Homology-based model of Bgl6 was constructed using the SWISS-MODEL [2] with the crystal structure of the BglA from *Thermotoga maritima* (PDB code: 1w3j). Visualization of the modeled structure was done using the program PyMOL (Delano Scientific, Palo Alto, CA, USA).

**Additional Figure S6.** High-Performance Liquid Chromatography (HPLC) analysis of the concentration of the celllobiose released from SCB. The analysis was carried out with a refractive index detector via a system equipped with a degassing system (LC-20A SHIMADZU, Japan) and a pump (SHIMADZU). A Shodex SUGAR SP0810 column (8.0×300 mm, 7 μm, Shodex, Japan) was used. HPLC-grade water was used as the mobile phase at a flow rate of 0.25 mL/min at 80 °C. The injection volume was 10 μL. Individual sugars in the mixtures were identified by comparison of the retention times with those of the standards and quantified from the peak area calibrated against sugar standards. The peaks were cellobiose, 33.237 min; glucose, 38.456 min.

**Additional Figure S7.** Time course of the cellobiose concentrations in the SCB hydrolysis. Celluclast 1.5 L (♦) was used alone as a control. Addition of Bgl6 (■) and mutant M3 (●) to Celluclast 1.5 L decreased the cellobiose cencentrations.

**Additional Figure S8.** Thin layer chromatography (TLC) analysis of the hydrolysis of hydrolysis products from SCB. M stranded for Marker, which is the mixture of 0.5 % (w/v) glucose and 0.5 % cellobiose (w/v). Line 1-8 represented the results of Celluclast 1.5L alone and referred to the hydrolysis time of 24, 48, 72, 92, 120, 168, 192 and 216 h, respectively; Line 9-16 represented the results of Celluclast 1.5L with mutant M3 and referred to the hydrolysis time of 24, 48, 72, 92, 120, 168, 192 and 216 h, respectively.

**Additional Figure S9.** Effects of glucose on the hydrolysis of pretreated SCB (10 %, w/v) by Celluclast 1.5 L (♦) alone and supplemented with mutant M3 (●). The initial glucose concentrations are 20 mM (A), 50 mM glucose (B), 100 mM (C), 200 mM (D), 300 mM (E) and 500 mM (F). Data represent the means of three experiments and error bars represent standard deviation.

- 1. **Figures**

**
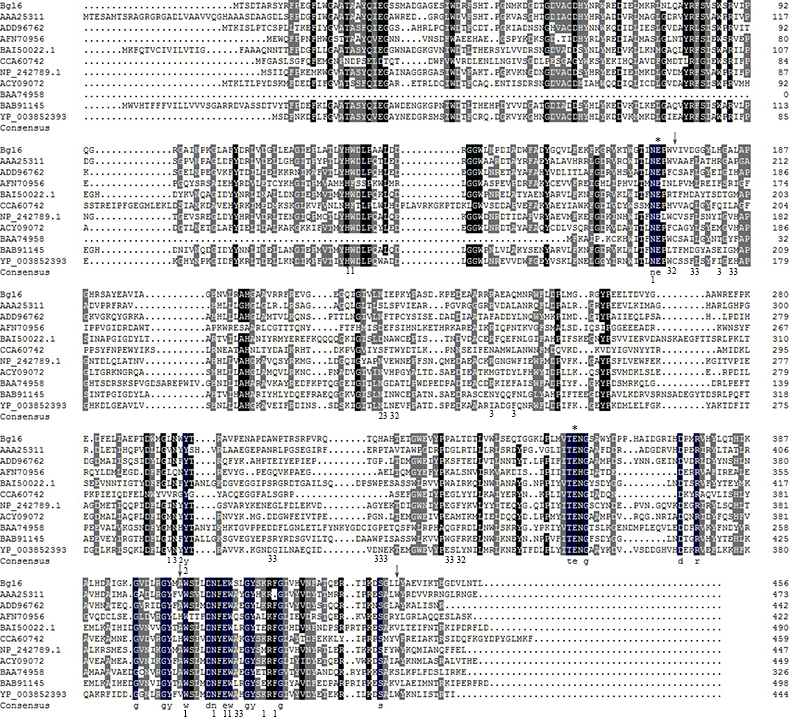
**

**Additional Figure S1.**


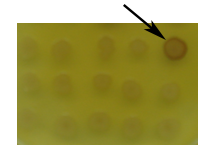


**Additional Figure S2.**

**A**


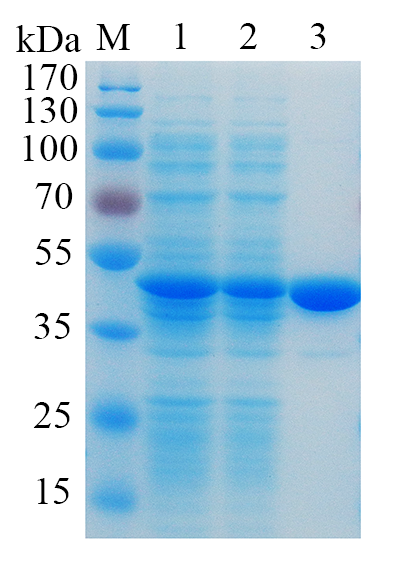


B


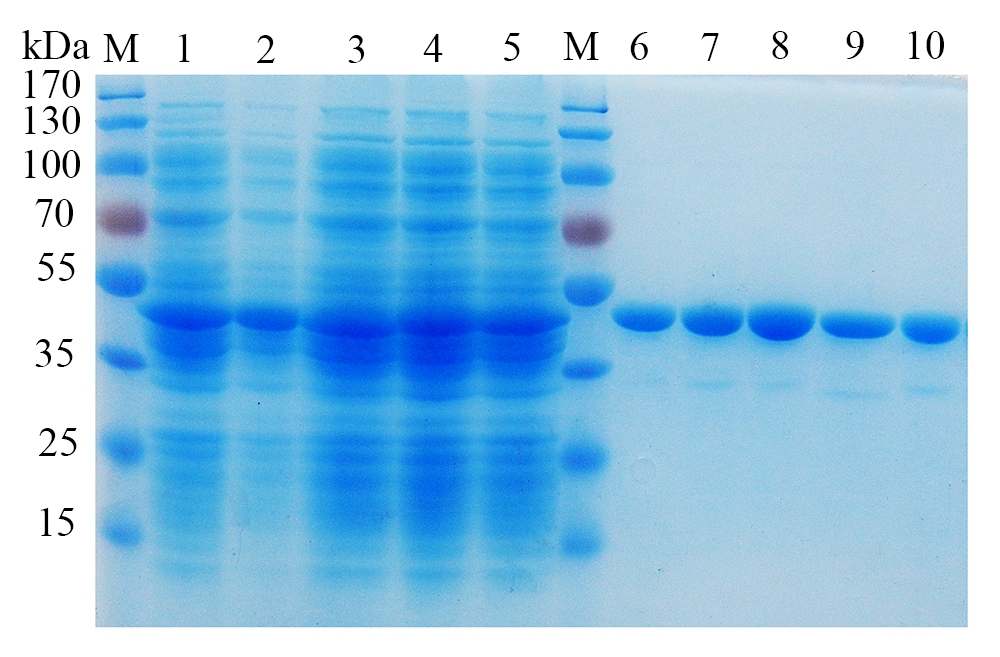


**Additional Figure S3.**


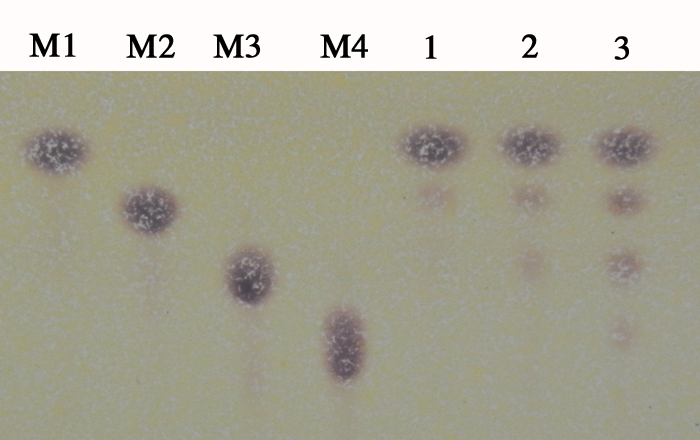


**Additional Figure S4.**

**A**

**
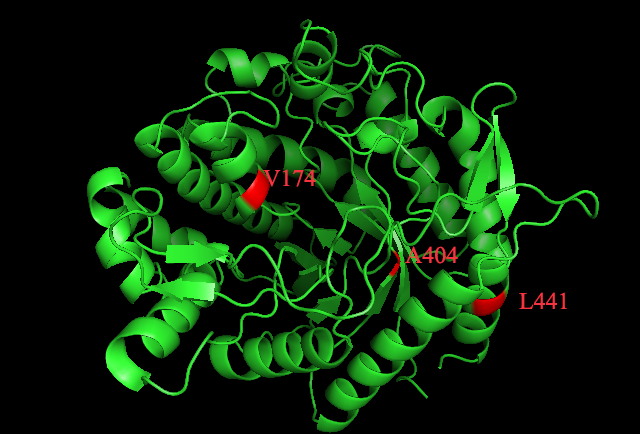
**

**B**

**
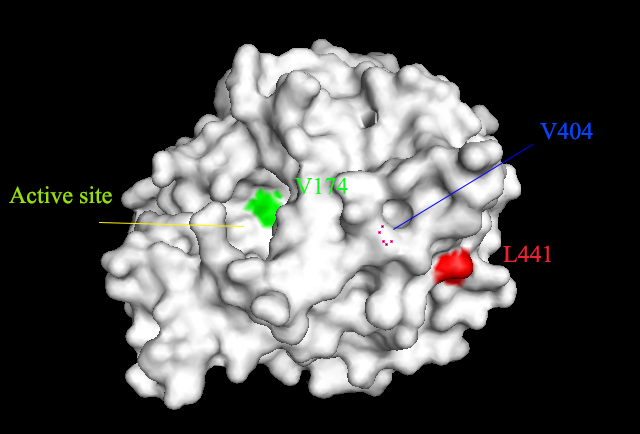
**

**Additional Figure S5**.


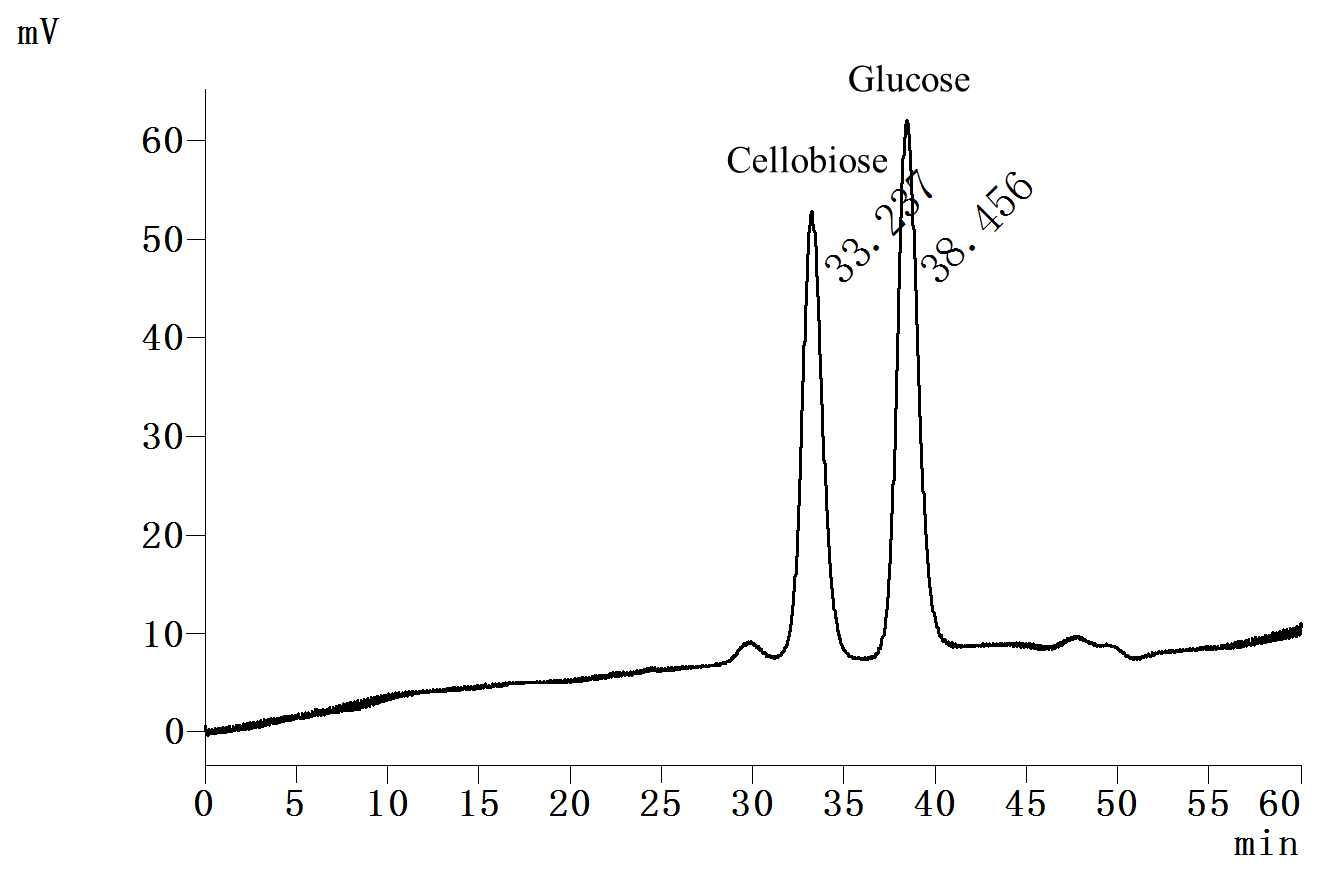


**Additional Figure S6**.


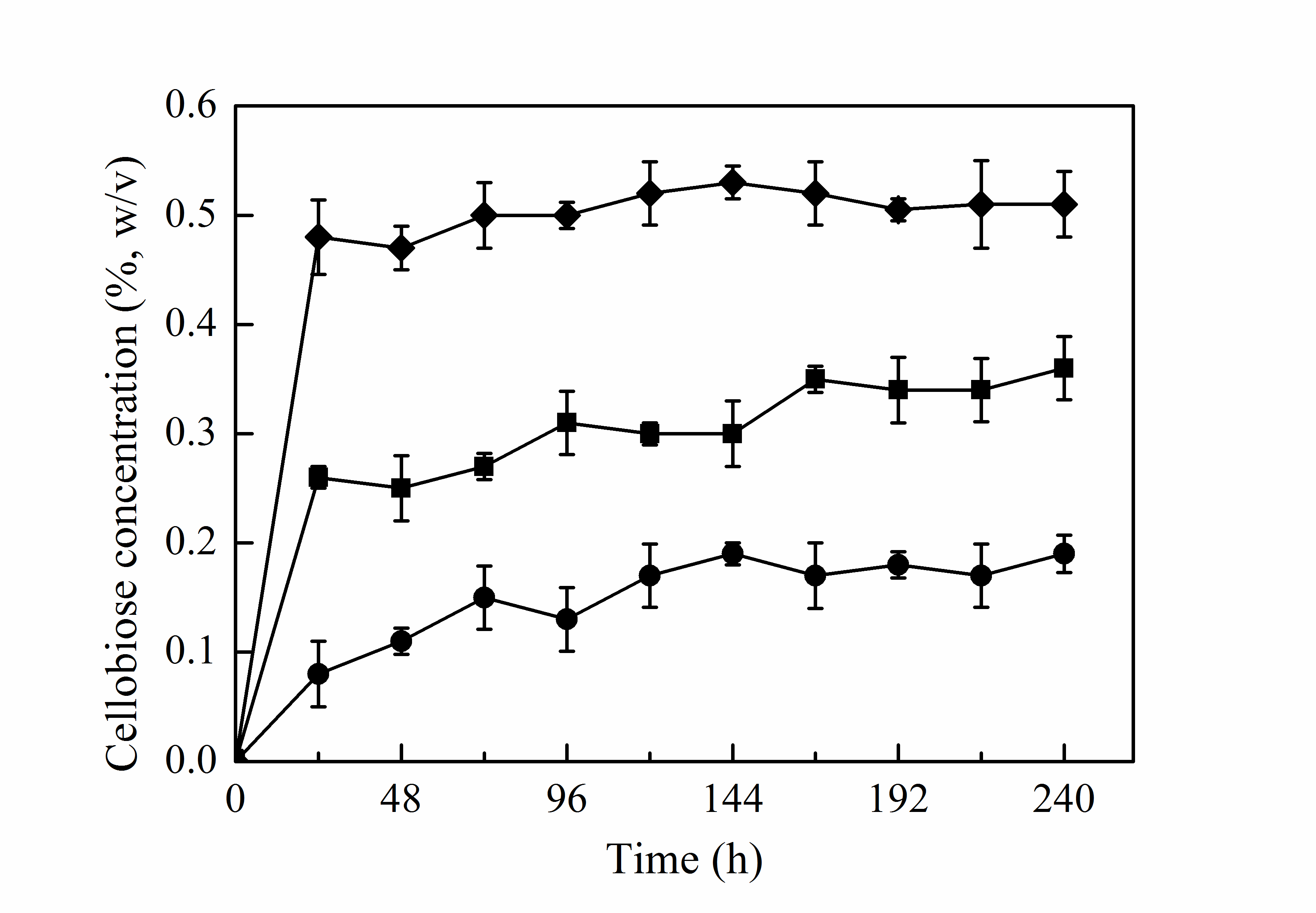


**Additional Figure S7.**


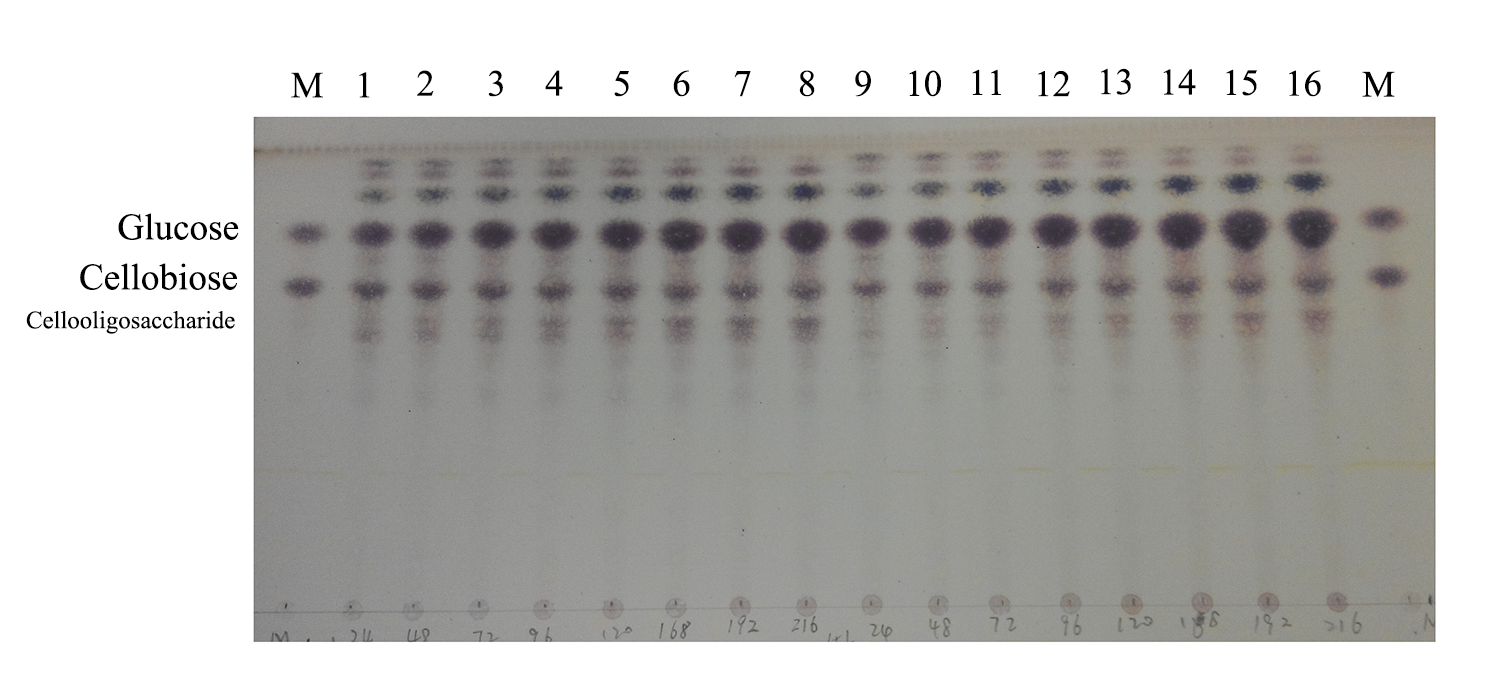


**Additional Figure S8.**


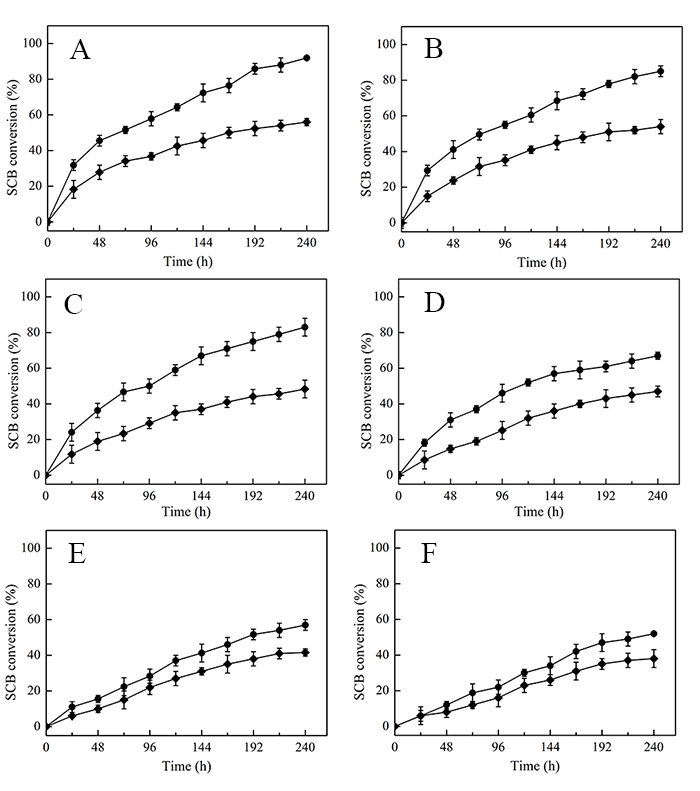


**Additional Figure S9**.

**Additional Table S1**

**Primers used to construct the mutants of Bgl6**

| Mutants | Primers |
| --- | --- |
| V174A | FP: GCGATCGTCGACGGCGGCTATCTGCACGG |
|  | RP: CCAGGGCTCGTTGATGGTGCCCCAG |
| V174C | FP: TGCATCGTCGACGGCGGCTATCTGCACGG |
|  | RP: CCAGGGCTCGTTGATGGTGCCCCAG |
| A404V | FP: GTTTGGTCGCTGCTCGACAATCTCG |
|  | RP: CATGTAGCCGCGCAGGTCGACGCCCT |
| L441F | FP: TTCTACGCCGAGGTCATCAAGACCCACG |
|  | RP: CAACCCCGAGTCCTTGATGGTCCGCTCC |

**References**

1. Lee HL, Chang CK, Jeng WY, Wang AH, Liang PH. Mutations in the substrate entrance region of β-glucosidase from *Trichoderma reesei* improve enzyme activity and thermostability. Protein Engineering, Design & Selection. 2012; 25(11):733-40.
2. Biasini M, Bienert S, Waterhouse A, Arnold K, Studer G, Schmidt T et al. SWISS-MODEL: modelling protein tertiary and quaternary structure using evolutionary information. Nucleic Acids Research. 2014; 42(W1):W252-W8.
